# Preparation and Desalination of Semi-Aromatic Polyamide Reverse Osmosis Membranes (ROMs)

**DOI:** 10.3390/polym15071683

**Published:** 2023-03-28

**Authors:** Haiyang Zhu, Bingbing Yuan, Yuchuan Li

**Affiliations:** 1 School of Materials Science and Engineering, Beijing Institute of Technology, Beijing 100081, China; 2 Lunan Research Institute of Beijing Institute of Technology, Tengzhou 277599, China; 3 School of Chemistry and Chemical Engineering, Key Laboratory of Green Chemical Media and Reactions, Ministry of Education, Collaborative Innovation Center of Henan Province for Green Manufacturing of Fine Chemicals, Henan International Joint Laboratory of Aquatic Toxicology and Health Protection, Henan Normal University, Xinxiang 453007, China

**Keywords:** cyclopentanecarbonyl chloride (CPTC), semi-aromatic, reverse osmosis membrane (ROM), desalination, preparation

## Abstract

Reverse osmosis membrane (ROM) technology has a series of advantages, such as a simple process, no secondary pollution, high efficiency, energy saving, environmental protection, and good separation and purification effects. High-performance semi-aromatic polyamide reverse osmosis membranes (ROMs) were prepared by interfacial polymerization (IP) of novel cyclopentanecarbonyl chloride (CPTC) and m-phenylenediamine (MPD) monomers. The surface morphology, hydrophilicity and charge of the ROMs were characterized by field-emission scanning electron microscopy (SEM), a contact angle tester and a solid-surface zeta potential analyzer. The effects of CPTC concentration, MPD concentration, oil-phase solvent type, IP reaction time and additive concentration on the performance of semi-aromatic polyamide ROMs were studied. SEM morphology characterization showed that the surface of the prepared polyamide ROMs presented a multinodal structure. The performance test showed that when the concentration of MPD in the aqueous phase was 2.5 wt.%, the concentration of sodium dodecylbenzene sulfonate (SDBS) was 0.2%, the residence time in the aqueous phase was 2 min, the concentration of CPTC/cyclohexane in the oil phase was 0.13 wt.%, the IP reaction was 20 s, the NaCl rejection rate of the semi-aromatic polyamide ROM was 98.28% and the flux was 65.38 L/m^2^·h, showing good desalination performance. Compared with an NF 90 commercial membrane, it has a good anti-BSA pollution ability.

## 1. Introduction

With the increasing shortage of human water resources, the problem of water pollution is now of great concern. Reverse osmosis membrane (ROM) technology has a series of advantages, such as a simple process, no secondary pollution, high efficiency, energy saving, environmental protection, and good separation and purification effects [1,2]. ROM technology was initially only applied to seawater desalination. With its increasingly obvious advantages in purification and desalination applications, ROM separation technology has also been gradually applied to environmental protection, the food and chemical industries, bionics, petroleum, medicine, the national defense and military industry and other fields [2,3,4,5,6]. With the continuous expansion of the application of membrane separation, there is also a demand for increased performance of reverse osmosis membranes (ROMs). Therefore, the development of new high-throughput, pollution-resistant ROMs is an important topic for membrane technology researchers [7].

A ROM is a composite structure usually composed of a top polyamide dense layer and a lower ultrafiltration membrane [8,9,10], wherein the top layer is formed by the interfacial polymerization of small molecular monomer trimethylene chloride (TMC) and m-phenylenediamine (MPD) with a thickness of 50–200 nm, and the lower layer is composed of a polysulfone porous support layer and polyester non-woven fabric to achieve high water permeability and good mechanical properties [11,12]. Generally speaking, the structure of the polyamide dense layer determines its desalting performance and antipollution performance. In view of this, researchers have adopted various methods, such as (1) the sue of other monomers in the process of interfacial polymerization [13,14,15,16,17,18], (2) surface coating [19,20,21,22], (3) membrane modification [23,24,25,26], (4) the introduction of nanoparticles into the water or oil phase [27,28], (5) the addition of soluble additives into the water or oil phase [29,30], etc., which have achieved different degrees of improvement in permeability and selectivity. For example, Liu et al. [15] prepared a chlorine-resistant reverse osmosis composite membrane on a polysulfone ultrafiltration membrane by two-step interfacial polymerization. The first interfacial polymerization involved the reaction of the crosslinking agent 5-chloroformyloxyisohalide chloride (CFIC) with 4-methylphenylenediamine (MMPD) to obtain an uncured primary poly(urethane) base membrane (CFIC–MMPD); then, the obtained base membrane was contacted with the functional secondary amine N, N′-dimethyl m-phenylenediamine (DMMPD) again to obtain a high-performance ROM. Ali Zain et al. [17] used a new trimethylene-1, 3, 6, 8-tetraacetyl chloride monomer to replace TMC to react with MPD to obtain a high-throughput ROM. The rejection rate of NaCl is about 96%, and the permeation flux is 9.2 LMH bar^−1^, which is better than that of an NF 270 commercial nanofiltration membrane.

Herein, we selected CPTC as the oil-phase monomer and reacted with MPD monomer in the water phase to conduct interfacial polymerization. The effects of CPTC concentration, MPD concentration, oil-phase solvent type, IP reaction time, additive type, etc., on the desalting performance of a semi-aromatic polyamide ROM were also studied in order to design and prepare an ROM with excellent performance.

## 2. Materials and Methods

### 2.1. Materials

Cyclopentaerythric anhydride (purity >98.0%), trimesoyl chloride (>98.0%), MPD (>98.0%) and dimethyl-aminopyridine (DMAP, >98.0%) were supplied by TCI Huacheng Industrial Development Co., Ltd. (Shanghai, China). Sulfoxide chloride (SOCl_2_, ≥99.0%), tetrachloroethane, chloroform (CH_3_Cl), bovine serum albumin (BSA, 97%, 66.43 kDa), N, N-dimethylacetamide (DMAc), n-heptane, concentrated hydrochloric acid, sodium hydroxide (NaOH), sodium chloride (NaCl), triethylamine (TEA, 99.5%), sodium dodecyl benzene sulfonate (SDBS), cyclohexane and Isopar G were provided by Sinopharm Chemical Reagents Co., Ltd., (Beijing, China). Camphor sulfonic acid (CSA, 99%) was supplied by Adamas Reagent Co., Ltd., (Shanghai, China). Polysulfone (PSF) ultrafiltration membrane was supplied by Toray Bluestar Membrane Co., Ltd., (Beijing, China). All of the chemicals were analytically pure and used as received. 

### 2.2. Instruments and Equipment

A an SU8010 field-emission scanning electron microscope (SEM), with an acceleration voltage of 5 kV and magnifications of 20.0 k and 50.0 k was supplied by Hitachi, Ltd. (Tokyo, Japan). An OCA20 contact angle tester (DataPhysics Instruments GmbH, Filderstadt, Germany); test method: sessile drop, 25 °C. Sur Pass 3 zeta potential analyzer for solid surfaces (Anton Paar GmbH, Graz, Austria); test conditions: 25 °C. Conductivity meter (Mettler-Toledo Instruments (Shanghai) Co., Ltd., Shanghai, China); test condition: 25 °C. Avanceiihd500 NMR spectrometer (Bruker, Mannheim, Germany).

### 2.3. Synthesis of Intermediate and Preparation of Sample

#### 2.3.1. Synthesis of CPTC

**Figure 1:**
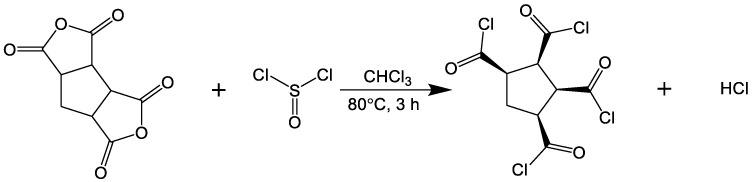
Synthesis of CPTC from cyclopentaerythric anhydride and sulfoxide chloride.

**Figure 2:**
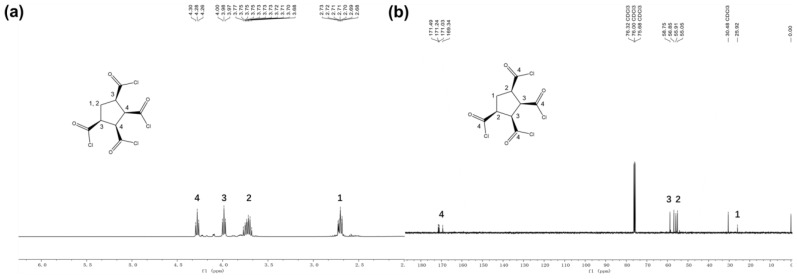
(**a**) ^1^H NMR spectra of the cis, cis, cis, cis-1,2,3,4-cyclopentane tetracarboxylic acid chloride (CPTC): ^1^H NMR (150 MHz, CDCl_3_) *δ*: 2.68–2.73, 3.68–3.77 (q, 2H, −CH_2_), 3.97–4.0 (d, 2H, −HC=O), 4.26–4.30 (t, 2H, −HC=O); (**b**) ^13^C NMR spectra of the cis, cis, cis, cis-1,2,3,4- cyclopentane tetracarboxylic acid chloride (CPTC): ^13^C NMR (150 MHz, CDCl_3_) *δ*: 25.92, 55.05–58.75, 169.34–171.49.

1,2,3,4-Cyclopentanetetracarboxylic Dianhydride was dried under vacuum at 65 °C for 8 h. As shown in Figure 1, the dried anhydride (20.0 g, 0.0925 mol), chloroform (50 mL) and sulfoxide chloride (40.45 g, 0.1943 mol) were stirred at 80 °C for 3 h. Chloroform and excess sulfoxide chloride were removed by vacuum distillation to obtain pure CPTC products (75.6%, 22.3 g). ^1^H NMR and ^13^C NMR spectra of CPTC are shown in Figure 2.

Figure 2a shows the 1H NMR spectra of CPTC. Peaks at 2.70–2.73 ppm (refers to shift 1 in the figure) and 3.68–3.77 ppm (refers to shift 2 in the figure) are assigned to -CH2 protons in the cyclopentane ring. Peaks at 3.97–4.00 ppm (refers to shift 3 in the figure) and 4.26–4.30 ppm (refers to shift 4 in the figure) are assigned to -CH protons in the cyclopentane ring. Figure 2b shows the 13C NMR spectra of CPTC. Peaks at 25.92 ppm (refers to shift 1 in Figure 2b) correspond to the -CH_2_ carbon. Peaks at 55.05–55.91 ppm (refers to shift 2 in the figure), 56.85–58.75 ppm (refers to shift 3 in the figure) and 171.03–171.49 ppm (refers to shift 4 in the figure) are assigned to -CH carbon in the cyclopentane ring.

#### 2.3.2. Preparation of Semi-Aromatic Polyamide ROM

**Preparation of aqueous amine solution:** The aqueous amine solution was composed of 100 mL deionized water, a certain amount of MPD, 2.09 g CSA, 0.91 g triethylamine (TEA), 0.1–0.3 g SDBS. Specifically, 100 mL deionized water was added to a conical flask; then, under a stirring condition, the MPD, CSA, TEA and SDBS were added one by one. Then, the prepared aqueous amine solution was standing for standby.

**Preparation of oil-phase CPTC solution:** The oil-phase CPTC solution consisted of 100 g of organic solvent (including n-heptane, cyclohexane and Isopar G) and a certain amount of CPTC. Specifically, 100 g of an organic solvent, such as n-heptane, was added to a conical flask; subsequently, a certain amount of CPTC was added under a magnetic mixer condition. The prepared oil-phase CPTC solution was stored under a dry condition and standby.

**Figure 3:**
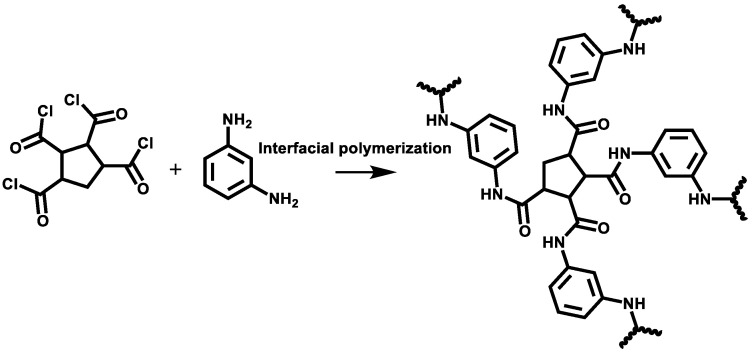
Preparation of semi-aromatic polyamide from CPTC and MPD.

**Semi-aromatic ROM prepared by IP from CPTC and MPD:** The PSF ultrafiltration membrane was immersed in the above-described aqueous amine solution for 3 min, and the excess solution was subsequently removed by an air knife with a pressure of 0.15 MPa. Then, the above-described PSF ultrafiltration membrane was soaked in the prepared oil-phase CPTC solution for a certain time to form an all-aromatic PA nanofilm. The reaction principle was shown in Figure 3. Then, the fresh polyamide nanomembrane was dried at 60 °C for 5 min and placed in deionized water for future use.

### 2.4. Characterizations

The CPTC monomer was characterized by NMR spectroscopy (Bruker, Mannheim, Germany). The surface morphology of the nanomembrane was characterized by scanning electron microscopy (SEM, SU8010, Hitachi, Tokyo, Japan). The sample was first laid on the sample plate and glued with conductive adhesive. The membrane sample was sprayed with gold for 30 s, then characterized by electron microscopy. Zeta potential measurement on the membrane surface: at 25 °C, 1 mM KCl was used as the electrolyte solution, and 0.05 mol/L HCl and NaOH solution was used as the pH regulator to adjust the relevant pH value to between 1 and 10 during the test; this was repeated 4 times to calculate the average value. The static contact angle of water in the membrane sample was measured by a contact angle tester, and five different positions were selected for each membrane sample.

**Performance and antipollution test:** The test conditions for the desalination performance test were as follows: operating pressure, 1.55 MPa; temperature, 25 °C; cross flow, 7.5 L/min; 2000 mg/L NaCl solution; test area, 28.12 cm^2^. The obtained polyamide membrane was stable for 1 h under the abovementioned conditions, and the data were recorded and calculated. The water flux and retention rate were obtained according to the following formula:

(1) $$\mathrm{water}\;\mathrm{flux}\;(\mathrm{kg}\;m^{-2}\;h^{-1})=M/\mathrm{At}$$

(2) $$\mathrm{rejection}\;\mathrm{rate}\;(\%)=\left({1–C}_{p}/C_{f}\right)\times 100\%$$

where M is the penetration weight (kg) of the semi-aromatic polyamide reverse osmosis membrane at the specified time (t, h); A is the effective membrane area (m^2^); and C_f_ and C_p_ are the conductivity (μs/cm) of the feed and permeate solution, respectively.

Anti-pollution performance test: In the process of pollution, the semi-aromatic polyamide ROM was first balanced at 1 MPa for 1 h, and the corresponding pure water flux (J_1_) was recorded. Then, the antifouling performance of the membrane was tested for 10 h in 1 MPa of 200 ppm BSA solution. The change data of the water flux of the prepared membranes were recorded every half hour. During the cleaning stage, the contaminated membranes were first cleaned with 0.1% NaOH solution at 1 MPa for 15 min. Then, the abovementioned membranes were again cleaned with pure water 3 times. Finally, the obtained pure water flux was recorded as J_2_. Cleaning efficiency (η) was calculated according to the following Formula (3):

(3) $$\eta (\%)=J_{2}/J_{1}\times 100\%$$

## 3. Results and Discussion

### 3.1. Analysis of Surface and Cross-Section Microstructure of ROM

**Figure 4:**
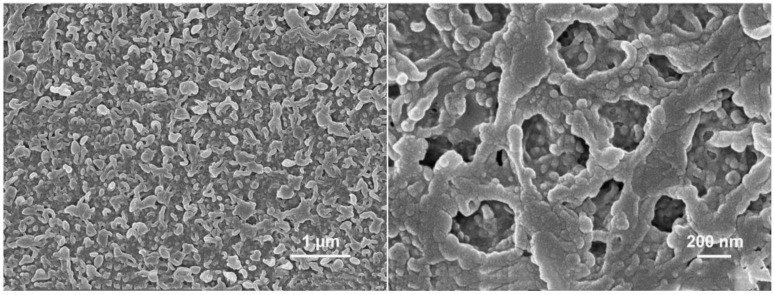
SEM images of semi-aromatic polyamide ROM.

**Figure 5:**
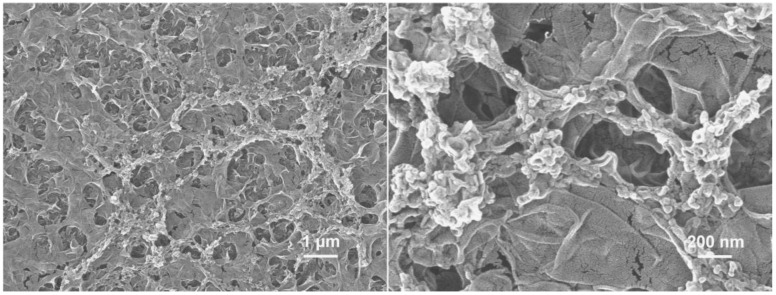
SEM images of fully aromatic polyamide ROM from NF 90 (DuPont).

**Figure 6:**
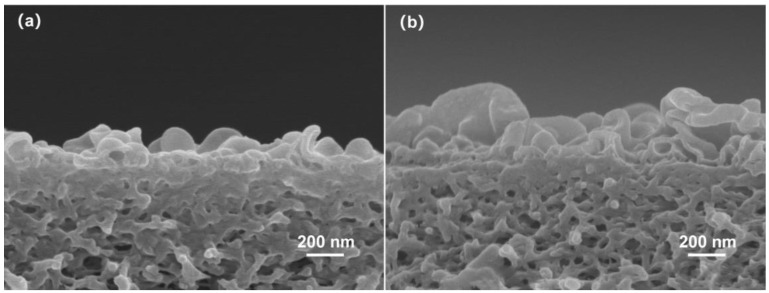
SEM images of semi-aromatic (**a**) and fully aromatic (**b**) polyamide ROM (from NF 90).

Figure 4 shows a SEM image of the semi-aromatic polyamide ROM prepared by CPTC and MPD, and Figure 5 shows a SEM image of the aromatic polyamide ROM prepared by trimethylene chloride (TMC) and m-phenylenediamine (MPD). Figure 4 shows that the surface of semi-aromatic polyamide ROM presents a micronodular structure with a low surface roughness. The aromatic polyamide ROM (TMC-MPD) presented in Figure 5 has a surface with a large number of lamellar stacking structures, which significantly increase the surface roughness. Figure 6a,b show the cross-section morphologies of the semi-aromatic (CPTC-MPD) and fully aromatic (TMC-MPD) polyamide membranes, demonstrating good homogeneity and compatibility with that of the surface investigation presented in Figure 4 and Figure 5. It is well known that the structure and morphology of IP polyamide membranes is mainly controlled by the diffusion reaction rate from the aqueous-phase amine monomer to the oil-phase acyl chloride monomer [31]. Compared with TMC as the oil-phase monomer, CPTC contains four acyl chloride functional groups. When CPTC reacts with MPD at the water–oil interface, the MPD monomer in the water phase shows a higher diffusion reaction rate because the interfacial polymerization between CPTC/TMC and MPD is a nucleophilic substitution reaction, and more nucleophilic groups such as acyl chloride functional groups can contribute to the nucleophilic substitution reaction in the direction of a positive reaction. In other words, compared with that of the TMC monomer, the CPTC monomer exhibits a faster nucleophilic reaction, so it can react with more MPD in the process of IP, forming more polar amide bonds. Moreover, considering the self-limited reaction characteristics of interfacial polymerization, the CPTC-MPD tends to exhibit a thinner nanofilm thickness. Because the CPTC monomer has more acyl chloride functional groups, the formation of the CPTC-MPD polyamide membrane occurs more rapidly, and the macromorphology shows a higher roughness.

### 3.2. Analysis of Surface Wettability of ROM

**Figure 7:**
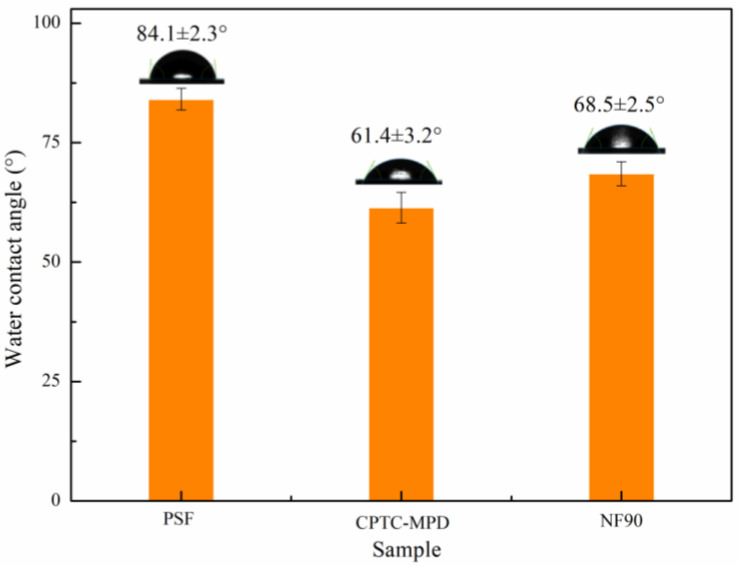
Water contact angle of the PSF ultrafiltration membrane and semi-aromatic (CPTC-MPD) and full-aromatic (NF 90) polyamide ROMs.

The water contact angle data presented in Figure 7 show that the water contact angles of the PSF ultrafiltration membrane and the semi-aromatic (CPTC-MPD) and aromatic (NF 90) polyamide ROMs are 84.1 ± 2.3°, 61.4 ± 3.2° and 68.5 ± 2.5°, respectively. The results show that the semi-aromatic polyamide ROM prepared by CPTC and MPD showed better hydrophilicity. There are two main reasons for the differences relative to the aromatic polyamide ROM: one is that when MPD reacts with CPTC, because CPTC contains four acyl chloride functional groups, MPD shows a fast nucleophilic reaction rate, so it can react with more MPD in the process of IP, forming more polar amide bonds and increasing the hydrophilicity. Secondly, among the three functional groups contained in the chain segment of the polyamide polymer, the amide bond, carboxyl group and amine group have a high dipole moment and hydration number. Therefore, directly increasing the number of amide bonds can increase the hydrophilicity of the polyamide membrane [32]. Combined with the above reasons, the semi-aromatic polyamide ROM has a lower water contact angle and better hydrophilicity than the aromatic polyamide ROM, which is also conducive to its good resistance to organic pollution in the antipollution performance test [33].

### 3.3. Surface Potential Analysis of ROM

**Figure 8:**
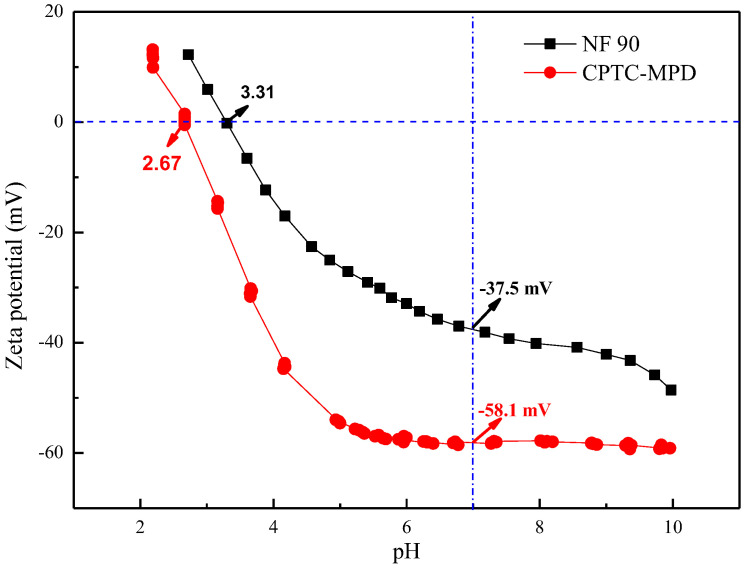
Zeta potential value of the semi-aromatic (CPTC-MPD) and fully aromatic (NF 90) polyamide ROMs. The electrolyte solution for zeta potential measurement was 1 mM KCl.

**Figure 9:**
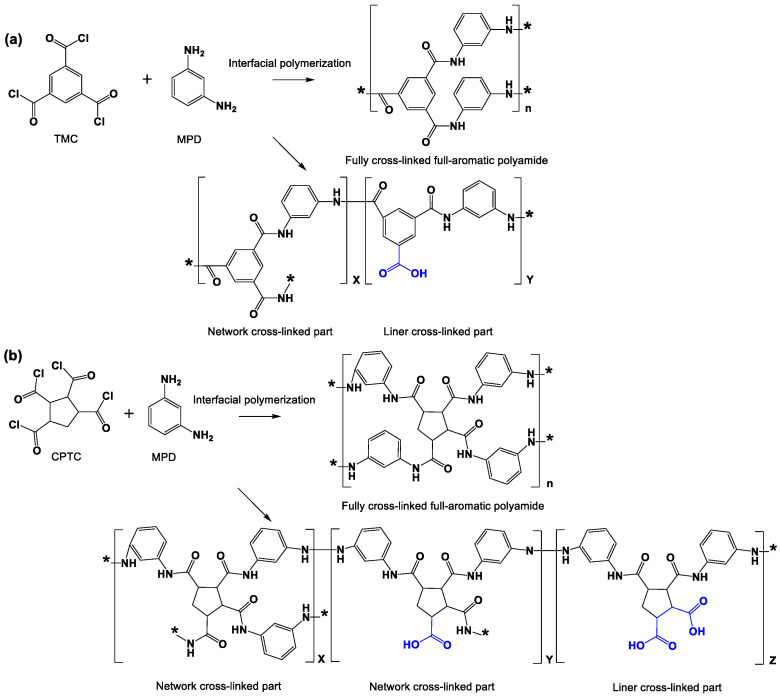
The reaction mechanism of (**a**) the fully aromatic (TMC-MPD) and (**b**) semi-aromatic (CPTCMPD) polyamide ROMs.

Figure 8 shows that the isoelectric points of semi-aromatic (CPTC-MPD) and aromatic (NF 90) polyamide ROMs are pH = 2.67 and pH = 3.31, respectively. When pH = 7, the membrane surface potentials of semi-aromatic (CPTC-MPD) and aromatic (NF 90) polyamide ROMs were −58.1 mV and −37.5 mV, respectively. These results show that compared with the aromatic polyamide ROM, the semi-aromatic polyamide ROM prepared by CPTC has more negative charges on its surface because CPTC contains four acyl chloride groups. In the process of interfacial polymerization, there are more residual acyl chloride functional groups that do not undergo nucleophilic substitution reaction with the amine group and are more easily hydrolyzed to carboxylic acid groups, resulting in more negative charges. As shown in Figure 9, the chemical structures of IP polyamide RO membranes comprise a fully crosslinked part and a linear part. The linear part of the fully aromatic (TMC-MPD) polyamide ROM has one carboxylic acid group. However, the linear part of the semi-aromatic (CPTC-MPD) polyamide ROM has two carboxylic acid groups, which is conducive to increasing the negative charge of the membrane surface.

### 3.4. Performance Analysis of Semi-Aromatic ROM

#### 3.4.1. Effect of MPD Concentration on Properties of Semi-Aromatic Polyamide Membrane

**Figure 10:**
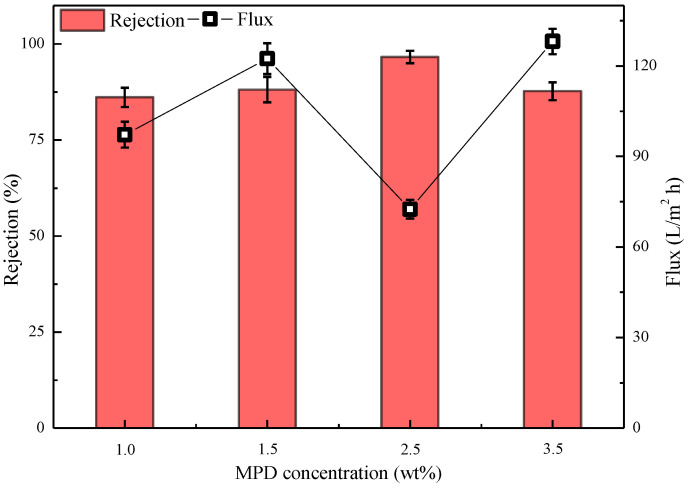
Effect of MPD concentration on the performance of the semi-aromatic polyamide membrane. Test conditions: 1.55 MPa, 2000 mg/L NaCl, 25 °C.

The membrane preparation conditions are shown in Figure 10: water-phase composition: 1.0%/1.5%/2.5%/3.5% MPD, 2.09% CSA, 0.91% TEA, 0.2% SDBS; oil-phase composition: cyclohexane solution of 0.11% CPTC; interfacial polymerization of 20 s. As shown in Figure 10, when the concentration of MPD is 2.5%, the semi-aromatic polyamide ROM has good retention and flux of 96.62 ± 1.6% and 72.53 ± 3.1 L/m^2^·h, respectively, which are superior to the performance of the semi-aromatic polyamide membrane prepared with concentrations of 1.0%, 1.5% and 3.5% in the aqueous phase.

It is well known that there is a “trade-off” phenomenon between the water flux and salt retention of polyamide nanomembranes, that is, high water flux and excellent retention often cannot be combined, and polyamide ROMs with high water flux usually exhibit low retention. The solution-diffusion model [34] describes the transport of water and salt through polymer membranes. The water flux through the polymer membrane is expressed by the following formula:

(4) $$J_{w}=\frac{P_{w}}{L}\frac{M_{w}}{\mathrm{RT}}\left(\Delta p-\Delta \pi \right)$$

where J_w_ is the water flux (g/cm^2^·s), P_w_ is the water permeability (cm^2^/s), M_w_ is the molecular weight of water (g/mol), L is the thickness of the nanomembrane (cm), R is the gas constant (83.1 cm^3^·bar/mol·K), T is the absolute temperature (K), Δp is the pressure difference on the membrane (bar) and ∆π is the osmotic pressure difference on the membrane (bar). 

Therefore, the water flux of polyamide ROM is inversely proportional to the membrane thickness. As shown in Figure 10, as the concentration of amine monomer in the aqueous phase increases from 1.0% to 2.5%, the thickness of the formed polyamide ROM gradually increases, and the defects of the ROM gradually increase, so the water flux decreases in turn. Meanwhile, the increase in the thickness causes the membrane to show a rising salt retention rate. However, when the concentration of amine monomer in the aqueous phase is further increased, the excessive concentration of MPD requires an increased concentration of acyl chloride monomer, and at this time, 0.11% CPTC is obviously insufficient. The ROM prepared under this condition shows defects, so the flux gradually increases, while the salt retention rate significantly decreases. 

On the basis of the above experimental data, the semi-aromatic polyamide ROMs were prepared with a 2.5% aqueous-phase concentration in subsequent experiments.

#### 3.4.2. Effect of CPTC Concentration on Properties of Semi-Aromatic Polyamide Membrane

**Figure 11:**
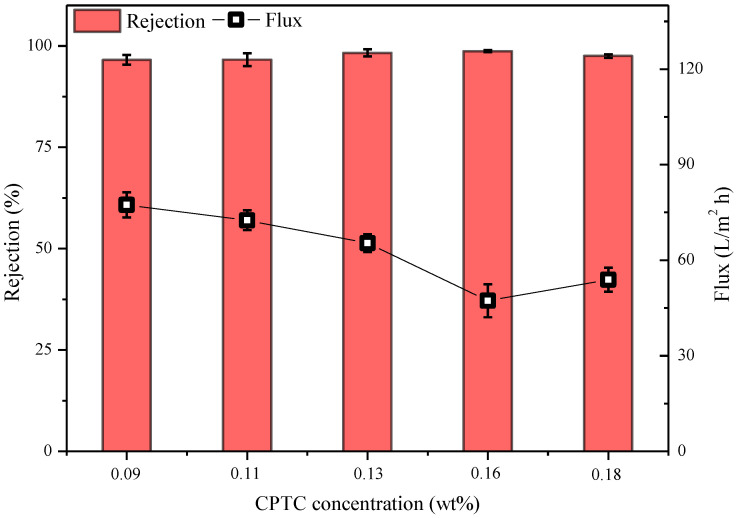
Effect of CPTC concentration on performances of semi-aromatic polyamide membranes. Test conditions: 1.55 MPa, 2000 mg/L NaCl, 25 °C.

The membrane preparation conditions corresponding to the data presented in Figure 11 were as follow: water-phase composition: 2.5% MPD, 2.09% CSA, 0.91% TEA, 0.2% SDBS; oil-phase composition: 0.09%/0.11%/0.13%/0.16%/0.18% cyclohexane solution of CPTC; interfacial polymerization time of 20 s. As shown in Figure 11, when the concentration of CPTC is 0.13% and 0.16%, the prepared semi-aromatic polyamide ROM has good retention and flux values of 98.28 ± 0.89% and 65.38 ± 2.8 L/m^2^·h, respectively, and 98.69 ± 0.24% and 47.28 ± 5.2 L/m^2^·h, which is superior to the performance of the semi-aromatic polyamide membrane with 0.09% and 0.18% concentrations of CPT. This is because with the gradual increase in the concentration of acyl chloride, the thickness of the prepared polyamide ROM also increases, so its water flux decreases. However, when the concentration of acyl chloride increases to 0.18%, the concentration of MPD in the water phase does not match the concentration of acyl chloride in the oil phase, so the NaCl retention rate decreases slightly [35]. In subsequent experiments, semi-aromatic polyamide ROMs were prepared with a 2.5% water phase concentration and a 0.13% oil-phase acyl chloride concentration.

#### 3.4.3. Effect of IP Time on Properties of Semi-Aromatic Polyamide Membrane

**Figure 12:**
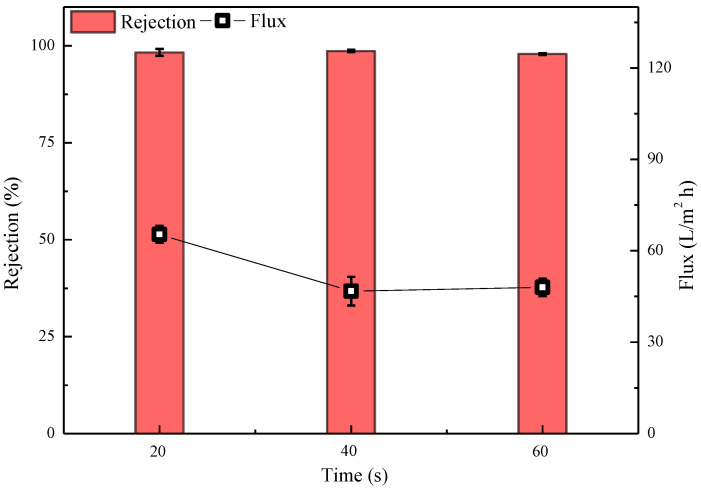
Effect of IP time on the performance of semi-aromatic polyamide membranes. Test conditions: 1.55 MPa, 2000 mg/L NaCl, 25 °C.

The membrane preparation conditions corresponding to the data presented in Figure 12 were as follows: water-phase composition: 2.5% MPD, 2.09% CSA, 0.91% TEA, 0.2% SDBS; oil-phase composition: 0.13% cyclohexane solution of CPTC; IP times of 20 s, 40 s and 60 s. As shown in Figure 12, when the IP time is 20 s and 40 s, the semi-aromatic prepared polyamide ROM has good retention and flux of 98.28 ± 0.89% and 65.38 ± 2.8 L/m^2^·h, and 98.64 ± 0.32% and 46.77 ± 4.7 L/m^2^·h, respectively, which is superior to the semi-aromatic polyamide membrane prepared with an IP time of 60 s because, according to the mechanism of interfacial polymerization membrane formation [36,37,38], the thickness of the formed polyamide ROM gradually increases with the extension of the interfacial polymerization time, so its water flux gradually decreases. In the following experiment, semi-aromatic polyamide ROM was prepared with a 2.5% aqueous-phase concentration, a 0.13% oil-phase acyl chloride concentration and an IP time of 20 s.

#### 3.4.4. Effect of SDBS Concentration on Properties of Semi-Aromatic Polyamide Membrane

**Figure 13:**
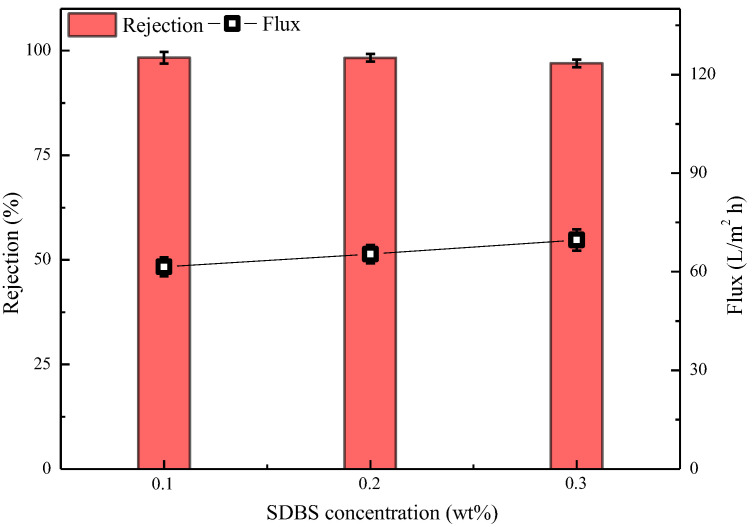
Effect of SDBS concentration on the performance of semi-aromatic polyamide membranes. Test conditions: 1.55 MPa, 2000 mg/L NaCl, 25 °C.

The membrane preparation conditions corresponding to the data presented in Figure 13 were as follows: water-phase composition: 2.5% MPD, 2.09% CSA, 0.91% TEA, 0.1%/0.2%/0.3% SDBS; oil-phase composition (cyclohexane solution of 0.13% CPTC); IP time of 20 s. As shown in Figure 13, when the concentration of SDBS is 0.1% and 0.2%, the semi-aromatic polyamide ROM prepared has good retention and flux of 98.32 ± 1.4% and 61.53 ± 2.9 L/m^2^·h, and 98.28 ± 0.89% and 65.38 ± 2.8 L/m^2^·h, respectively, which is better than the performance of the semi-aromatic polyamide membrane prepared with 0.3% SDBS. The reason is that SDBS, as a surfactant, can increase the wettability of the aqueous amine monomer solution on the surface of the hydrophobic ultrafiltration membrane, causing more aqueous amine monomers to soak into the pores of the ultrafiltration membrane [39] and reducing the diffusion reaction rate of the aqueous amine monomers to the acyl chloride monomers in the oil phase. The resulting polyamide reverse osmosis membrane is thinner, and the water flux increases [40]. In subsequent experiments, semi-aromatic polyamide ROMs were prepared with a 2.5% aqueous-phase concentration, a 0.2% SDBS concentration, a 0.13% oil-phase acyl chloride concentration and an IP time of 20 s. 

#### 3.4.5. Effect of the Type of Oil-Phase Solvent on Properties of Semi-Aromatic Polyamide Membrane

**Figure 14:**
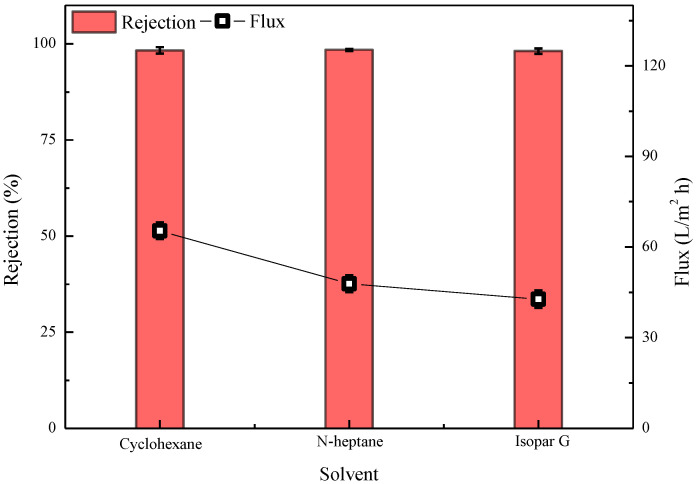
Effect of the type of oil-phase solvent on the performance of semi-aromatic polyamide membranes. Test conditions: 1.55 MPa, 2000 mg/L NaCl, 25 °C.

The membrane preparation conditions corresponding to the data presented in Figure 14 were as follows: water-phase composition: 2.5% MPD, 2.09% CSA, 0.91% TEA, 0.1%/0.2%/0.3% SDBS; oil-phase composition: 0.13% cyclohexane solution of CPTC/0.13% heptane solution of CPTC/0.13% Isopar G solution of CPTC; and IP time of 20 s. As shown in Figure 14, when the oil-phase solvent is cyclohexane, the semi-aromatic prepared polyamide ROM has good retention and flux of 98.28 ± 0.89% and 65.38 ± 2.8 L/m^2^·h, respectively, which is superior to the performance of the ROM prepared with n-heptane and Isopar G as solvents. This is because cyclohexane is more volatile than n-heptane and Isopar G, forming a dense layer with relatively loose structure, which is conducive to achieving a higher flux.

In summary, when the concentration of MPD is 2.5%, the concentration of SDBS is 0.2%, the concentration of CPTC is 0.13%, the IP time is 20 s and the oil-phase solvent is cyclohexane, the prepared semi-aromatic polyamide nanomembrane can achieve excellent retention and flux.

### 3.5. Anti-Pollution Performance Analysis of Semi-Aromatic Polyamide ROM

**Figure 15:**
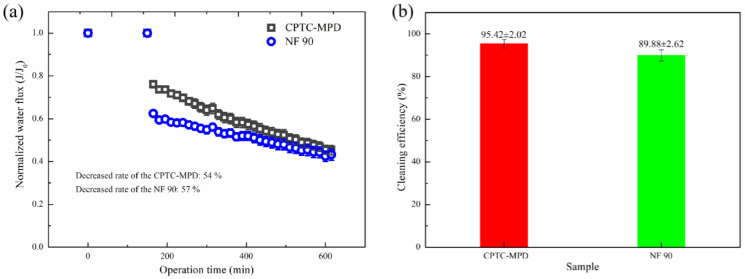
(**a**) Normalized water flux decline as a function of time and cleaning efficiency (**b**) for the CPTC-MPD and TMC-MPD PA membranes. The model feed solution used for the fouling experiment was as follows: 200 ppm BSA solution, 1 MPa. The experimental temperature was 25 °C.

Anti-pollution performance is an important feature of ROMs in practical industrial applications. In Figure 15a, BSA is used as a model organic pollutant to evaluate the pollution behavior of CPTC-MPD and NF 90 (TMC-MPD) polyamide ROMs. With the deposition of BSA on the surface of the separation layer, the water flux of the prepared polyamide membrane decreased slowly. It is noteworthy that compared with the NF 90 membrane, the normalized water flux of CPTC-MPD membrane decreases slowly. During the operation time of 0–600 min, the standardized water flux reduction rates of CPTC-MPD and NF 90 are 54% and 57%, respectively. These data show that compared with the aromatic polyamide ROM, the semi-aromatic polyamide ROM with better hydrophilicity and lower roughness has better anti-BSA pollution ability. On the other hand, in order to further evaluate the antifouling performance of the membrane, the cleaning efficiency was measured and calculated. As shown in Figure 15b, the average cleaning efficiency of the CPTC-MPD membrane is 95.42%, which is higher than that of the NF 90 membrane (89.88%).

Generally speaking, the antipollution performance of polyamide ROMs is related to the surface roughness, charge and hydrophilicity of the membrane, as well as the type of pollutants. For negatively charged BSA pollutants in the feed solution with pH = 7, high negative surface charge can significantly reduce the deposition of pollutants on the membrane surface [41,42] because the adsorption of negatively charged BSA on the negatively charged membrane surface is inhibited by electrostatic repulsion. The surface is negative at pH = 7 with a zeta potentials of −58.1 mV and −37.5 mV for the NF 90 (TMC-MPD) membrane. The results show that the CPTC-MPD membrane showed greater electrostatic repulsion than the NF 90 (TMC-MPD) membrane in inhibiting BSA deposition. On the other hand, the CPTC-MPD membrane shows higher roughness and better hydrophilicity than the NF 90 (TMC-MPD) membrane. Therefore, compared with the NF 90 (TMC-MPD) membrane, the CPTC-MPD membrane is more well-suited to reduce the adhesion of BSA on the surface of the membrane, showing better antipollution performance. Therefore, we conclude that the enhanced antipollution ability of the CPTC-MPD membrane against BSA can mainly be attributed to low roughness, significant hydrophilicity and a more negative surface charge, which is conducive to reducing the adhesion between BSA and the membrane surface [43,44]. We reasonably estimate that if a CPTC-MPD membrane is applied to water softening, the current membrane life and desalination efficiency will be improved.

### 3.6. Long-Term Operational Stability of the Prepared CPTC-MPD ROM

**Figure 16:**
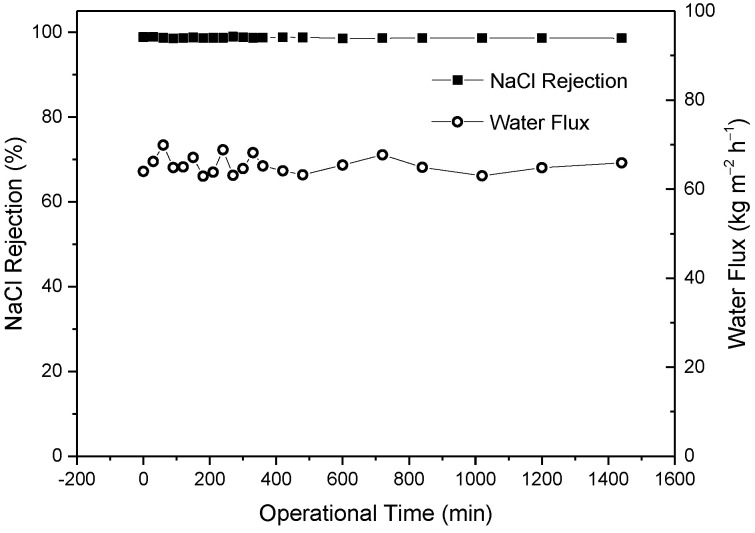
The long-term stability and durability of the fabricated CPTC-MPD polyamide ROM. Test conditions: 1.55 MPa, 2000 mg/L NaCl, 25 °C.

As shown in Figure 16, during a continuous 24 h test, the fabricated CPTC-MPD polyamide ROM showed stable NaCl rejection (from 98.56–98.94%) and water flux (from 62.9 to 69.89 kg m^−2^ h^−1^), which demonstrates that the CPTC-MPD nanofilm has a good crosslinked structure. This result demonstrates that the fabricated CPTC-MPD ROM has a potential for further industrialized desalination of brackish water.

### 3.7. Comparison of Desalination Performance between CPTC-MPD and Membranes Reported in the Literature

**Figure 17:**
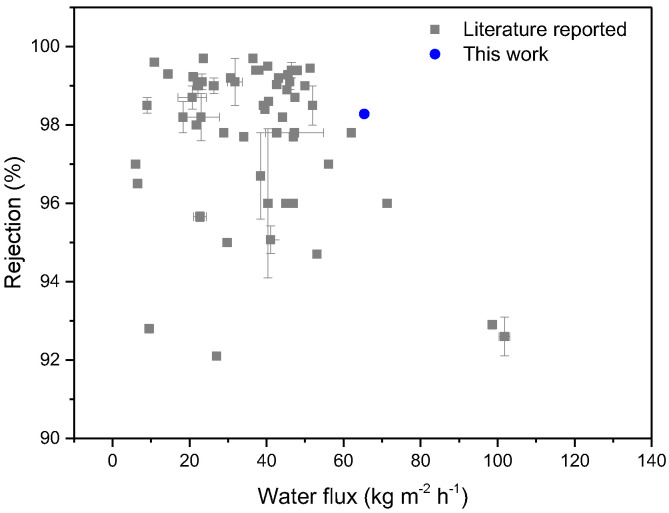
Comparison of NaCl rejection and water flux between polyamide membranes fabricated via CPTC-MPD and membranes reported in the literature fabricated by TMC and MPD.

We compared NaCl rejection and water flux between the polyamide membranes fabricated via CPTC-MPD and membranes reported in the literature prepared by MPD-TMC. As shown in Figure 17, the semi-aromatic polyamide ROM made by CPTC-MPD showed a good balance between NaCl rejection and water flux, exceeding most of fully aromatic polyamide ROMs reported in the literature. The related data are shown in Table S1, Supporting Information. All the membranes reported in the literature were fabricated by TMC and MPD. For example, the molecular layer-by-layer RO membrane exhibited a rejection of 98.7 ± 0.3% for 2000 mg/L NaCl, and the related water flux was 20.7 ± 3.7 kg m^−2^ h^−1^ [45].

## 4. Conclusions

In this thesis, a novel acyl chloride monomer, CPTC, was successfully designed and synthesized. A novel semi-aromatic polyamide nanomembrane was prepared by the reaction of CPTC with MPD. SEM electron microscopy showed that the surface of the semi-aromatic polyamide nanomembrane had a uniform nodular structure and low roughness. The water contact angle and zeta potential indicate that it has good hydrophilicity and high surface charge. The desalting performance test showed that the semi-aromatic polyamide ROM has a good retention rate (>98.28%) and high flux (65.38 L/m^2^·h). An antipollution test showed that the semi-aromatic polyamide ROM has good anti-BSA pollution ability due to its good hydrophilicity and low roughness caused by uniform nodule structure. In summary, the prepared CPTC-MPD polyamide membranes have an excellent water flux and high NaCl rejection rate compared to classic TMC membranes such as NF 90. Moreover, the CPTC-MPD polyamide ROM has lower roughness than that of classic TMC membranes. Hence, during in household and industrial brackish water market, CPTC-MPD membranes are a preferred alternative to conventional TMC-based polyamide membranes. Furthermore, novel acid chloride can be synthesized by the facile acyl chloride reaction, and by making the scaling-up of the interfacial polymerization process feasible, the CPTC-MPD membrane can be conveniently industrialized for real-world applications.

## Data Availability

Not applicable.

## Supplementary Materials

The following supporting information can be downloaded at: https://www.mdpi.com/article/10.3390/polym15071683/s1, Table S1: Comparison of NaCl rejection from the polyamide membranes fabricated via CPTC-MPD, and literatures reported membranes that are fabricated by TMC and MPD.
