# Supplementary material for: Preparation and Desalination of Semi-Aromatic Polyamide Reverse Osmosis Membranes (ROMs)
Source: Polymers (Basel). 2023 Mar 28;15(7):1683. doi: 10.3390/polym15071683 (PMC10096747; doi:10.3390/polym15071683)
Supplement: Supplementary file 1 [file polymers-15-01683-s001.zip › polymers-2261371-supplementary.pdf]

Title Preparation and Desalination of Semi-Aromatic Polyamide Reverse Osmosis Membranes (ROMs)

Haiyang Zhu [1,2], Bingbing Yuan [3,*] and Yuchuan Li [1,*]

[1] School of Materials Science and Engineering, Beijing Institute of Technology, Beijing 100081, China
[2] Lunan Research Institute of Beijing Institute of Technology, Tengzhou 277599, China
[3] School of Chemistry and Chemical Engineering, Key Laboratory of Green Chemical Media and Reactions, Ministry of Education, Collaborative Innovation Center of Henan Province for Green Manufacturing of Fine Chemicals, Henan International Joint Laboratory of Aquatic Toxicology and Health Protection, Henan Normal University, Xinxiang 453007, China
* Correspondence: yuanbingbing@htu.edu.cn (B.Y.); liyuchuan@bit.edu.cn (Y.L.); Tel.: +86-010-68915822 (Y.L.)

Table S1. Comparison of NaCl rejection from the polyamide membranes fabricated via CPTC-MPD, and literatures reported membranes that are fabricated by TMC and MPD.

Membrane	NaCl Rejection (%)	Water flux (kg m^{-2} h^{-1})
ROM-3	97	56.1
CD-TFC-250	99.0 ± 0.3	22.2 ± 1.24
TFC-B	95.66 ± 0.12	22.7 ± 1.7
PIP-DHBA-DHBA	99.1 ± 0.2	46.04
ZIF-67/PA	99.28	45.57
PA-IL	99.2	30.7
PA-$MMP_{0.05}$	94.7	53.1
FBN-PA	97.8	62
TFC-1D-3	96.7 ± 1.1	38.44
TFN-Ns-0.06	96	71.3
TFC	97.7	47
PA/POSS	98	21.7
HPP-ZIF-8-RO	99	50
ROM-DEP-0.5	98.2	44.18
IPSA-3	99.2	43.09
TFC-M	98.9	45.32
KRO-1	99.6	10.9
DSC-DPPE	99.5 ± 0.1	40.3
pPa2CNs/PA	97.7	34.1
PMABSA/TMC	98.2±0.4	18.29±0.17
Ag-NPs	99.0 ± 0.2	26.35
RO-stress-rinse	99.39	48
GO-ZnO-RO	99.3	14.4
rGO/TiO2/RO	99.45	51.3
TFN-GOQD/AP50	98.4	39.6

PA04 TFN	92.1	27
MWCNT/RO	97.8	28.9
L-0.2	99.03	42.63
0.1%-CNC-TFN	97.8	47.25 ± 7.5
MoS2-TFN	92.9	98.6
DSC	99.1 ± 0.6	31.8 ± 1.9
CNC-TFC	99.1 ± 0.2	23.25
IP@FI-0.05	96.0 ± 1.9	40.35
$mLbL_{15}$-PA	98.7 ± 0.3	20.7 ± 3.7
TFC-pH10.3	99.4 ± 0.1	37.2
TFN-ZIF-8	98.5 ± 0.5	51.93
TMC/MPD mLbL	96.5	6.51
g-C3N4 NS PA/CN100	99.23	20.95
TFN MR-I	96	47
PA (nano zeolite)	~95	29.79
MPD/TMC	92.8	9.51
PPD/TMC	97	6.0
MPD/TMC	96	45
TFN-mGO	99.7	23.6
TFN	98.7	47.36
RO-PSbPDz	99	26.2
PA-GO	99.7	36.5
RO-Ti3C2Tx	98.5	39.22
PA-TFC-DMSO-0.3	99.4 ± 0.2	46.5
TFC-6	97.8	42.63
RO-0.015	99.4	38.05
P(mPDTA)	98.2 ± 0.6	23.0 ± 4.8
PA/BHPF	95.07 ± 0.35	41.08 ± 1.1
PA-ZIF-8	98.6	40.5

XLE	92.6 ± 0.5	101.8 ± 1.4
NF 90	83.8 ± 0.3	125.4 ± 1.0
SW30HR	98.5 ± 0.2	9.0 ± 0.8
CPTC-TAEA	98.28	65.38
